# The Use of Amberlite Adsorbents for Green Chromatography Determination of Volatile Organic Compounds in Air

**DOI:** 10.1155/2012/728143

**Published:** 2012-07-17

**Authors:** Luis Juan-Peiró, Anne Bernhammer, Agustin Pastor, Miguel de la Guardia

**Affiliations:** ^1^Department of Analytical Chemistry, University of Valencia, Research Building, 50th Dr. Moliner Street, 46100 Burjassot, Spain; ^2^Institute of Inorganic and Analytical Chemistry, Johannes Gutenberg University Mainz, Duesbergweg 10-14, 55128 Mainz, Germany

## Abstract

Passive samplers have been widely used for volatile organic compounds determination. Following the green chemistry tendency of the direct determination of adsorbed compounds in membrane-based devices through using head space direct chromatography analysis, this work has evaluated the use of Amberlite XAD-2, XAD-4, and XAD-16 adsorbents as a filling material for passive samplers. Direct analysis of the membranes by HS-GC-MS involves a solvent-free method avoiding any sample treatment. For exposed membranes, recoveries ranged from 10% to 203%, depending on the compound and adsorbent used. The limit of the detection values ranged from 1 to 140 ng per sampler. Acceptable precision and sensitivity levels were obtained for the XAD resins assayed.

## 1. Introduction

Volatile organic compounds (VOCs) are a wide group of chemicals, which can be emitted to the atmosphere from different sources [1]. Toxicity of some of them has been reported during the last years, with some VOCs classified as harmful compounds to human health [2, 3]. These compounds have a potential role in the formation of photooxidants and they can be considered as the reason for olfactory pollution [4]. In the case of benzene, this compound exhibits a cancer risk and causes aplastic anemia and polycythemia, with toluene, ethylbenzene, and xylenes being important noncarcinogenic agents [5]. On the other hand, the VOCs and specially those halogenated compounds contribute to global warming, stratospheric ozone depletion and tropospheric ozone formation. Therefore, it is important to establish a control and to monitor the air concentration of VOCs in both working areas and private places.

Air sampling techniques commonly used to evaluate the quality of indoor and outdoor air are based on the use of active or passive samplers, with low-flow active ones being also used in order to evaluate long-term deploying times. In general, active samplers provide direct measurements of the concentration of contaminants per m^3^. However, their use is costly and involves complex sampling systems due to the use of pumps when passive samplers offer cheap and easy alternatives [7, 8]. Passive samplers are commonly used for the determination of VOCs in air [9], with the semi-permeable membrane devices (SPMDs) being one of the most used among them [8]. SPMDs consist of low-density polyethylene (LDPE) lay-flat tube filled with triolein and sealed at both ends and have been used as an adsorbent of hydrophobic compounds in water and air. Our research team developed a modified version of these passive samplers, in which triolein was substituted by a solid phase or a combination of solid phases introduced inside a semipermeable membrane, thus improving the SPMD sampling range, due to their ability for retaining a wide range of compounds with different physicochemical properties [10]. These devices were registered under the name of Versatile, Easy, and Rapid Atmospheric Monitors (VERAM), and previous results obtained with a solid filling of activated carbon and florisil were successfully applied to different studies of sampling and analysis of air quality, with several compounds being determined such as pyrethroids, VOCs, or pesticides [3, 11–13]. Determination of VOCs using VERAM devices and analysis of samples through head space gas chromatography with mass spectrometry detection (HS-GC-MS) could be considered as a green method since it involves a solvent-free sample manipulation and direct determination, thus avoiding the accumulation of toxics residues and the consequent environmental side-effects [14]. However, additional efforts are required to increase the capability of these devices as chemical catchers based on the use of alternative adsorbents and membranes.

**Figure 1:**
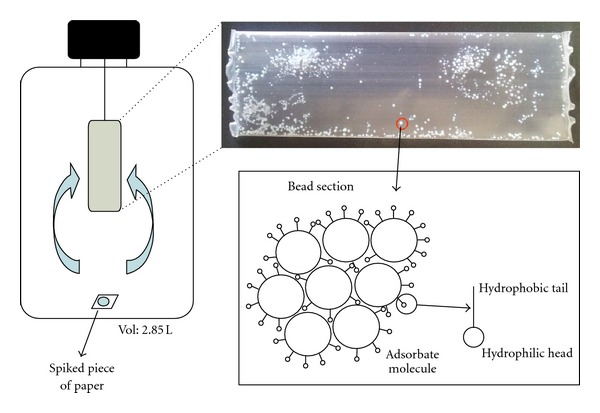
Diagram of the sampler and sampling process. Inset: structure of an Amberlite XAD-2 resin bead.

The aim of the present work has been to use Amberlite XAD resins to filling LDPE membranes, in order to compare the possible advantages in front of the classical VERAM samplers. XAD resins are hydrophobic nonionic crosslinked polymers provided as white beads, which are widely used to adsorb organic compounds as it can be seen in the inset of Figure 1. These resins have been applied as XAD-based passive samplers for routing air monitoring of persistent organic pollutants (POPs) and other semivolatile organic compounds (SVOCs) [15]. Depending on their physicochemical properties, such as surface area or pore diameter, XAD resins can modify their adsorption properties (see Table 1), and Lee et al. made a comparison between XAD-2, XAD-4, and XAD-16 resins for the retention of vapour-phase PAHs [16]. 

Therefore, this study has been focused on the use of XAD-2, XAD-4, and XAD-16 adsorbents as alternative filling materials of VERAM devices, comparing the results found with the previous ones obtained with activated carbon and florisil (ACFL).

## 2. Materials and Methods

### 2.1. Chemicals

Studied VOC standards and hexadecane were obtained from Scharlau (Barcelona, Spain), Fluka Chemie (Steinheim, Germany), Merck (Darmstadt, Germany), and Aldrich (Steinheim, Germany). Working standard solutions were prepared by dilution of a stock solution (3.85% (w/w) VOCs standard stock solution prepared by mixing 1 g of all considered analytes) in hexadecane. Hexadecane was employed due to its high boiling point. Activated carbon from Panreac (Barcelona, Spain), Florisil from Acros (Geel, Belgium), and Amberlite XAD-2, XAD-4, and XAD-16 from Sigma Aldrich (Steinheim, Germany), were employed as solid phases for adsorption studies. 2.85 ± 0.01 L volume glass containers employed were appropriately decontaminated and cleaned up using a 5 min nitrogen flow before being used.  Table 2 shows the list of studied compounds as well as their measurement parameters.

### 2.2. Equipment

A Finnigan Trace Gas Chromatograph (Waltham, MS, USA) with a low bleed column HP (30 m × 0,32 mm × 0,25 *μ*m), equipped with a Thermo-Finnigan HS2000 head-space injector and coupled to a Finnigan Polaris Q ion trap mass spectrometer detector was used for VOCs determination. Glass vials with an internal volume of 10 mL, capped with PTFE/butyl rubber seals, were employed for HS measurements. LPDE lay-flat tubing, 2,9 cm wide, was obtained from Garci-plast (Barcelona, Spain), and Rovebloc sealer (Barcelona, Spain) was employed to heat-seal the membranes.

### 2.3. Preparation of Samplers

Preparation of samplers was carried out according to previous published method [3]. LDPE lay-flat tubing was cut into 10 cm segments. The segments were cleaned in hexane overnight and air-dried. One end was heat sealed before introducing the corresponding filling: 50 mg of Amberlite XAD-2; 50 mg of Amberlite XAD-4; 50 mg of Amberlite XAD-16; or 50 mg of florisil and 5 mg of activated carbon.

XAD resins were soaked in deionized water/acetone (1 : 1) for 10 min inside an ultrasound water bath, followed by acetone soaking and drying. Then the adsorbents were soaked in dichloromethane/acetone (1 : 1) for another 10 minutes, mesh-sieved, and thoroughly dried.

Furthermore, all solid phases were heated up to 145°C for two hours to reduce the amount of VOC contamination.

The filled membranes were wrapped in separate aluminium foils and stored in a closed vessel at −20°C to avoid their contamination before use. After deployment, the devices were rolled, placed inside 10 mL HS vials, capped hermetically with PTFE/butyl rubber seals, and stored at −20°C until the HS-GC-MS measurement.

Calibration curves were obtained from membranes spiked with 5 *μ*L of VOC hexadecane standard solutions at 7 concentration levels ranging from 15 ng to 1500 ng together with 1500 ng of d8-toluene and 1500 ng of d12-cyclohexane as internal standards.

### 2.4. Adsorption Studies

Adsorption studies were performed by deploying membranes filled with XAD-2, XAD-4, XAD-16 and active carbon-florisil inside closed 2.85 ± 0.01 L glass bottles, where 2 cm^2^ pieces of filter paper spiked with 150 ng of VOCs were previously introduced (see Figure 1). Sampling was done at room temperature for 72 h. Blank measurements were carried out in the same way by deploying each membrane inside glass bottles for 72 h without adding VOCs. All the deployments were done by triplicate.

After exposure, membranes were transferred into 10 mL HS vials, and after adding 1500 ng of d8-toluene and 1500 ng of d12-cyclohexane, both of them prepared in hexadecane, vials were hermetically capped.

### 2.5. Spiked Membranes

Membranes filled with the different adsorbent materials evaluated were spiked with 150 ng of VOC standard mixture, in order to compare their HS-GC-MS signals with those obtained for the deployed membranes and to determine the recovery percentage of each considered compound. These membranes were directly introduced into the HS vials after adding 1500 ng of d8-toluene and 1500 ng of d12-cyclohexane as internal standards. 

### 2.6. HS-GC-MS Direct Determination

Desorption of VOCs was carried out at 145°C during 10 min, with a syringe temperature of 145°C. A volume of 0,1 mL was then injected into the GC (split 1 : 8) at 200°C. Helium (99,99% purity) was used as carrier gas with a constant flow of 1,3 mL/min. Initial temperature of GC oven was set at 40°C and held for 8 min, then it was increased 20°C min^−1^ up to 200°C, and finally held for 2 min. Transfer line and ion source temperatures were 280 and 250°C, respectively. Detection was done in Full Scan mode ranging from 40 to 260 m/z with an electron impact ionization of 70 eV.

## 3. Results and Discussion

### 3.1. The Influence of Adsorbents on VOCs HS-GC-MS Determination

**Figure 2:**
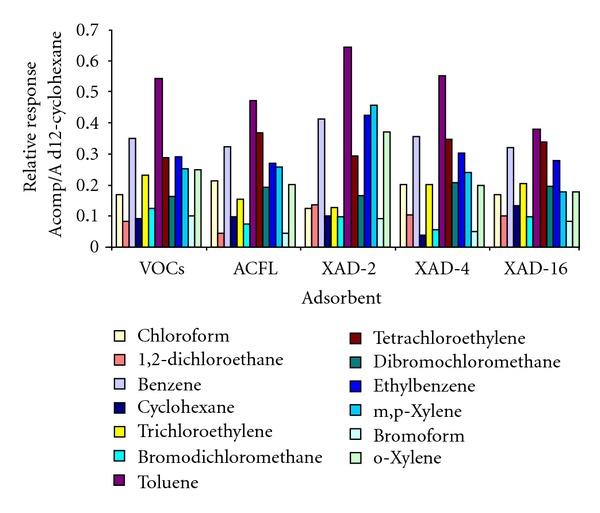
Relative response of VOCs determined by HS-GC-MS in membranes spiked with 150 ng of each considered compound (*n* = 3).

In order to check the response of XAD resins under chromatography conditions, membranes filled with XAD-2, XAD-4, and XAD-16 were spiked with 150 ng of VOCs standard mixture and were analysed by HS-GC-MS. After blank correction, results found were compared with those obtained with active carbon-florisil classical VERAM samplers (see Figure 2). Analytical response of most of the compounds desorbed from XAD resins is similar to that obtained from ACFL filled samplers, which suggests a similar behaviour of resins under chromatography conditions. The direct measurement of VOCs solutions through HS-GC-MS is also indicated in the figure for comparative purposes regarding the capability of the membranes to retain VOCs in the selected measurement conditions.

**Figure 3:**
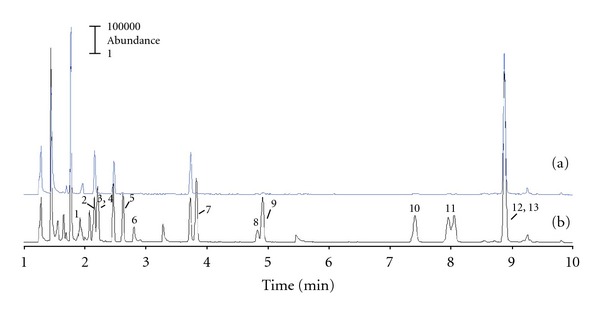
Total ion chromatogram obtained for a membrane filled with 50 mg of XAD-2 (a) and a membrane filled with 50 mg of XAD-2 spiked with 1500 ng of VOC standard mixture (b). Note: peaks correspond to Chloroform (1), 1,2-Dichloromethane (2), Benzene (3), Cyclohexane (4), Trichloroethylene (5), Bromodichloromethane (6), Toluene (7), Dibromochloromethane (8), Tetrachloroethylene (9), Ethylbenzene (10), m,p-Xylene (11), Bromoform (12), and o-Xylene (13).

Figure 3 shows peak identification for all the considered compounds in the total ion chromatogram obtained from a membrane filled with XAD-2 spiked with VOCs, and from it the easy identification of all compounds can be appreciated. The chromatogram obtained for a passive sampler blank clearly shows that Amberlite adsorbents can be alternative solid phases for VERAM devices.

### 3.2. The Capability of XAD for VOCs Adsorption

**Figure 4:**
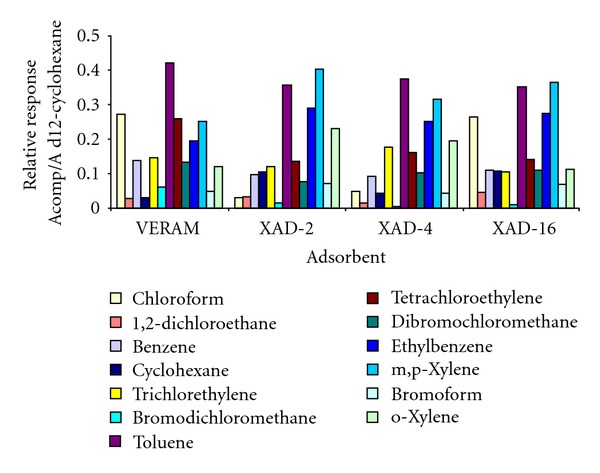
Relative response of VOCs in membranes deployed for 72 h and filled with different adsorbent Amberlite materials (*n* = 3).

As said before, adsorbent materials can retain a certain range of compounds depending on their properties. Tests of XAD adsorption were carried out in order to evaluate the capability of the resins for extraction VOCs from air as LDPE membrane fillings.  Figure 4 shows the relative adsorption for all the compounds under study of XAD-2, XAD-4, and XAD-16 samplers exposed during 72 h, and after blank corrections most of the compounds present a similar response.

As can be observed in Table 3, most of the recovery results obtained from Amberlite resins are similar to the well-known carbon ACFL filled samplers, ranging from 10% to 203% for XAD adsorbents. Recovery percentages were calculated by triplicate by comparison of signals obtained from deployed membranes for 72 h with the responses corresponding to spiked membranes. 

### 3.3. Analytical Figures of Merit

Table 4 shows analytical parameters obtained for the determination of VOCs by HS-GC-MS after retention in LDPE membranes filled with different adsorbents. Precision of the method was evaluated from RSD values, with results lower than 22% in all cases for 3 independent determinations. Limits of detection of XAD resins were calculated as follows: LOD = 3 *S*_blank_/*b*, where *b* is the slope of the calibration curve and *S*_blank_ is the standard deviation (SD) of 3 independent measurements of a passive sampler spiked with 15 ng of VOCs and were compared with those obtained for VERAM samplers in previous studies [3]. The slope of the calibration curve, *b*, gives the sensibility of the method for each compound. XAD-2 shows best limits of detection among the resins ranging from 1 to 140 ng per sampler.

## 4. Conclusions

Amberlite XAD-2, XAD-4, and XAD-16 are alternative filling materials for LPDE-based passive samplers in order to do VOCs determination. Results obtained in this preliminary study evidence similar adsorption properties for Amberlite resins than for ACFL-filled membranes, which have been widely used with success in the past but providing very low limit of detection values. Although XAD-2 seems to show better analytical parameters, VOCs adsorption results obtained for amberlite resins are similar for all of them, and it could be used in future studies.

## Figures and Tables

**Table 1:** Physicochemical properties of Amberlite resins [6].

Resin	CAS number	DRY density (versus wet) (g/mL)	Surface area (m^2^/g)	Mean pore size (Angstroms)	Particle size (mesh)	Pore vol. (mL/g)
XAD-2	9060-05-3	1.07 (1.02)	330	90	20 to 60	0.65
XAD-4	37380-42-0	1.08 (1.02)	725	50	20 to 60	0.98
XAD-16	104219-63-8	1.08 (1.02)	900	100	20 to 60	1.82

**Table 2:** GC-MS measurement parameters of the studied VOCs.

Compound	*R*_*t*_ (min)	Measurement ions (m/z)	Boiling point (^°^C)	*P*_*v*_ 20^°^C (mmHg)	Log *K*_OA_
Chloroform	1.93	93 + 85	62	159	2.80
1,2-Dichloroethane	2.16	62 + 64	84	387	2.78
Cyclohexane-d12	2.17	64 + 96	81	94	2.65
Benzene	2.20	78 + 77	80	101	2.78
Cyclohexane	2.23	56 + 84	81	95	2.74
Trichloroethylene	2.63	132 + 130	87	75	2.99
Bromodichloromethane	2.81	85 + 83	87	50	3.06
Toluene-d8	3.70	98 + 100	111	28	3.30
Toluene	3.83	91 + 92	111	29	3.31
Dibromochloromethane	4.84	127 + 129	120	76	3.59
Tetrachloroethylene	4.92	166 + 164	121	14	3.48
Ethylbenzene	7.41	91 + 106	136	10	3.74
m,p-Xylene	7.96	91 + 106	139	8	3.78
Bromoform	8.90	173 + 175	149	5	4.06
o-Xylene	8.96	91 + 106	144	7	3.91

Note: *R*_*t*_: Retention time; *P*_*v*_: vapour pressure; *K*_OA_: octanol-air partition coefficient.

**Table 3:** VOCs recovery values expressed in % of signals found for deployed membranes as compared with spiked ones obtained for different adsorbents (*c* = 150 ng · sampler^−1^).

Compound	Recovery %			
	Veram	XAD-2	XAD-4	XAD-16
Chloroform	128	26	24	156
1,2-Dichloroethane	63	24	16	45
Benzene	43	24	26	35
Cyclohexane	31	107	111	80
Trichloroethylene	95	94	88	52
Bromodichloromethane	84	15	11	10
Toluene	89	55	68	92
Tetrachloroethylene	70	47	46	41
Dibromochloromethane	70	47	50	56
Ethylbenzene	73	68	83	99
m,p-Xylene	98	88	131	203
Bromoform	105	77	87	84
o-Xylene	60	62	97	64

**Table 4:** Analytical features of HS-GC-MS determination of studied VOCs for each adsorbent evaluated.

Compound	Veram		XAD-2			XAD-4			XAD-16		
	RSD	LOD	RSD	LOD	*b *	RSD	LOD	*b *	RSD	LOD	*b *
	(%)	ng · sampler^−1^	(%)	ng · sampler^−1^	(×10^−3^)	(%)	ng · sampler^−1^	(×10^−3^)	(%)	ng · sampler^−1^	(×10^−3^)
Chloroform	5	50	13	31	1.96	9	30	2.04	9	37	2.03
1,2-Dichloroethane	9	50	23	30	0.91	12	73	0.85	38	13	0.87
Benzene	7	10	9	1	3.77	10	4	3.66	10	4	3.72
Cyclohexane	5	50	10	130	0.82	3	130	0.70	12	39	1.08
Trichloroethylene	6	50	5	3	2.17	4	4	1.99	12	23	1. 95
Bromodichloromethane	6	50	7	10	1.32	4	95	1.03	14	140	1.11
Toluene	6	10	5	11	6.48	47	90	7.61	22	58	5.49
Tetrachloroethylene	5	10	4	17	3.22	7	41	2.96	18	29	2.85
Dibromochloromethane	6	50	4	15	2.50	7	28	1.71	19	19	1.64
Ethylbenzene	9	10	3	3	3.76	12	6	3.22	13	5	2.88
m,p-Xylene	13	10	6	14	3.28	11	12	2.79	17	10	2.44
Bromoform	15	50	1	2	0.99	15	8	0.65	19	7	0.75
o-Xylene	13	10	2	8	2.85	14	21	2.47	14	114	2.13

Note: RSD %: relative standard deviation for 3 independent determinations (*c* = 1500 ng · sampler^−1^); LOD: Limit of detection expressed in ng · sampler^−1^; *b*: slope of calibration curve expressed in relative units.

## References

[1] U.S. Environmental Protection Agency An Introduction to Indoor Air Quality. http://www.epa.gov/.

[2] Ly-Verdú S, Esteve-Turrillas FA, Pastor A, de la Guardia M (2010). Determination of volatile organic compounds in contaminated air using semipermeable membrane devices. *Talanta*.

[3] Ly-Verdú S, Esteve-Turrillas FA, Pastor A, de la Guardia M (2010). A passive sampling-based analytical strategy for the determination of volatile organic compounds in the air of working areas. *Analytica Chimica Acta*.

[4] Kim KH, Shon ZH, Kim MY, Sunwoo Y, Jeon EC, Hong JH (2008). Major aromatic VOC in the ambient air in the proximity of an urban landfill facility. *Journal of Hazardous Materials*.

[5] Durmusoglu E, Taspinar F, Karademir A (2010). Health risk assessment of BTEX emissions in the landfill environment. *Journal of Hazardous Materials*.

[6] Product Information Amberlite XAD polymeric resins. http://www.sigmaaldrich.com/.

[7] Batterman S, Metts T, Kalliokoski P, Barnett E (2002). Low-flow active and passive sampling of VOCs using thermal desorption tubes: Theory and application at an offset printing facility. *Journal of Environmental Monitoring*.

[8] Esteve-Turrillas FA, Yusà V, Pastor A, de la Guardia M (2008). New perspectives in the use of semipermeable membrane devices as passive samplers. *Talanta*.

[9] Kot-Wasik A, Zabiegała B, Urbanowicz M, Dominiak E, Wasik A, Namieśnik J (2007). Advances in passive sampling in environmental studies. *Analytica Chimica Acta*.

[10] Pastor A, de la Guardia M, Esteve-Turrillas FA

[11] Esteve-Turrillas FA, Ly-Verdú S, Pastor A, de la Guardia M (2009). Development of a versatile, easy and rapid atmospheric monitor for benzene, toluene, ethylbenzene and xylenes determination in air. *Journal of Chromatography A*.

[12] Esteve-Turrillas FA, Pastor A, De La Guardia M (2005). Determination of pyrethroid insecticide residues in vegetable oils by using combined solid-phases extraction and tandem mass spectrometry detection. *Analytica Chimica Acta*.

[13] Sanjuán-Herráez D, Rodríguez-Carrasco Y, Juan-Peiró L, Pastor A, de la Guardia M (2011). Determination of indoor air quality of a phytosanitary plant. *Analytica Chimica Acta*.

[14] Armenta S, Garrigues S, de la Guardia M (2008). Green Analytical Chemistry. *TrAC Trends in Analytical Chemistry*.

[15] Tuduri L, Millet M, Briand O, Montury M (2012). Passive air sampling of semi-volatile organic compounds. *TrAC Trends in Analytical Chemistry*.

[16] Lee JJ, Huang KL, Yu YY, Chen MS (2004). Laboratory retention of vapor-phase PAHs using XAD adsorbents. *Atmospheric Environment*.

